# Phenotypic recapitulation and correction of desmoglein-2-deficient cardiomyopathy using human-induced pluripotent stem cell-derived cardiomyocytes

**DOI:** 10.1093/hmg/ddab127

**Published:** 2021-05-05

**Authors:** Mikio Shiba, Shuichiro Higo, Takumi Kondo, Junjun Li, Li Liu, Yoshihiko Ikeda, Yasuaki Kohama, Satoshi Kameda, Tomoka Tabata, Hiroyuki Inoue, Satoki Nakamura, Maki Takeda, Emiko Ito, Seiji Takashima, Shigeru Miyagawa, Yoshiki Sawa, Shungo Hikoso, Yasushi Sakata

**Affiliations:** Department of Cardiovascular Medicine, Osaka University Graduate School of Medicine, Suita, Osaka 565-0871, Japan; Department of Cardiovascular Medicine, Osaka University Graduate School of Medicine, Suita, Osaka 565-0871, Japan; Department of Medical Therapeutics for Heart Failure, Osaka University Graduate School of Medicine, Suita, Osaka 565-0871, Japan; Department of Cardiovascular Medicine, Osaka University Graduate School of Medicine, Suita, Osaka 565-0871, Japan; Department of Cardiovascular Surgery, Osaka University Graduate School of Medicine, Suita, Osaka 565-0871, Japan; Department of Design for Tissue Regeneration, Osaka University Graduate School of Medicine, Suita, Osaka 565-0871, Japan; Department of Cardiovascular Surgery, Osaka University Graduate School of Medicine, Suita, Osaka 565-0871, Japan; Department of Design for Tissue Regeneration, Osaka University Graduate School of Medicine, Suita, Osaka 565-0871, Japan; Department of Pathology, National Cerebral and Cardiovascular Center, Suita, Osaka 564-8565, Japan; Department of Cardiovascular Medicine, Osaka University Graduate School of Medicine, Suita, Osaka 565-0871, Japan; Department of Cardiovascular Medicine, Osaka University Graduate School of Medicine, Suita, Osaka 565-0871, Japan; Department of Cardiovascular Medicine, Osaka University Graduate School of Medicine, Suita, Osaka 565-0871, Japan; Department of Cardiovascular Medicine, Osaka University Graduate School of Medicine, Suita, Osaka 565-0871, Japan; Department of Medical Therapeutics for Heart Failure, Osaka University Graduate School of Medicine, Suita, Osaka 565-0871, Japan; Department of Medical Biochemistry, Osaka University Graduate School of Medicine, Suita, Osaka 565-0871, Japan; Department of Cardiovascular Surgery, Osaka University Graduate School of Medicine, Suita, Osaka 565-0871, Japan; Department of Cardiovascular Surgery, Osaka University Graduate School of Medicine, Suita, Osaka 565-0871, Japan; Department of Medical Biochemistry, Osaka University Graduate School of Medicine, Suita, Osaka 565-0871, Japan; Department of Cardiovascular Surgery, Osaka University Graduate School of Medicine, Suita, Osaka 565-0871, Japan; Department of Cardiovascular Surgery, Osaka University Graduate School of Medicine, Suita, Osaka 565-0871, Japan; Department of Cardiovascular Medicine, Osaka University Graduate School of Medicine, Suita, Osaka 565-0871, Japan; Department of Cardiovascular Medicine, Osaka University Graduate School of Medicine, Suita, Osaka 565-0871, Japan

## Abstract

Desmoglein-2, encoded by *DSG2*, is one of the desmosome proteins that maintain the structural integrity of tissues, including heart. Genetic mutations in *DSG2* cause arrhythmogenic cardiomyopathy, mainly in an autosomal dominant manner. Here, we identified a homozygous stop-gain mutations in *DSG2* (c.C355T, p.R119X) that led to complete desmoglein-2 deficiency in a patient with severe biventricular heart failure. Histological analysis revealed abnormal deposition of desmosome proteins, disrupted intercalated disk structures in the myocardium. Induced pluripotent stem cells (iPSCs) were generated from the patient (R119X-iPSC), and the mutated *DSG2* gene locus was heterozygously corrected to a normal allele via homology-directed repair (HDR-iPSC). Both isogenic iPSCs were differentiated into cardiomyocytes [induced pluripotent stem cell-derived cardiomyocytes (iPSC-CMs)]. Multielectrode array analysis detected abnormal excitation in R119X-iPSC-CMs but not in HDR-iPSC-CMs. Micro-force testing of three-dimensional self-organized tissue rings (SOTRs) revealed tissue fragility and a weak maximum force in SOTRs from R119X-iPSC-CMs. Notably, these phenotypes were significantly recovered in HDR-iPSC-CMs. Myocardial fiber structures in R119X-iPSC-CMs were severely aberrant, and electron microscopic analysis confirmed that desmosomes were disrupted in these cells. Unexpectedly, the absence of desmoglein-2 in R119X-iPSC-CMs led to decreased expression of desmocollin-2 but no other desmosome proteins. Adeno-associated virus-mediated replacement of *DSG2* significantly recovered the contraction force in SOTRs generated from R119X-iPSC-CMs. Our findings confirm the presence of a desmoglein-2-deficient cardiomyopathy among clinically diagnosed dilated cardiomyopathies. Recapitulation and correction of the disease phenotype using iPSC-CMs provide evidence to support the development of precision medicine and the proof of concept for gene replacement therapy for this cardiomyopathy.

## Graphical Abstract

**Graphical Abstract:**
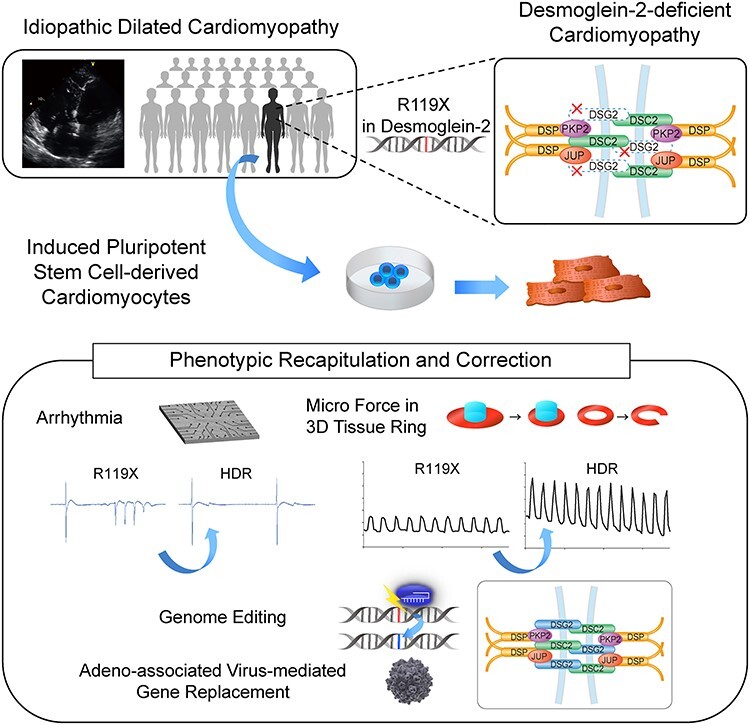

## Introduction

Dilated cardiomyopathy (DCM) is a life-threatening, intractable disease characterized by ventricular dilation and thinning. The prognosis of advanced heart failure resulting from DCM remains poor, even under the maximum pharmacological and non-pharmacological therapies. The etiology of DCM is wide-ranging, and recent advances of high-throughput sequencing technologies have shown that genetic mutation is the dominant cause of DCM in both familial and sporadic cases (1,2). The cellular functions of the causative genes are diverse and involve the sarcomere, Z-disk/cytoskeleton, ion flux, nucleus, cell membrane and intercalated disk (1). Desmosomes, junctional structures located at intercalated disks between cells, maintain the structural integrity of tissues, including heart (3). Four desmogleins (*DSG1*–*DSG4*) and three desmocollins (*DSC1*–*DSC3*) comprise the desmosome cadherin genes. Desmoglein-2 and desmocollin-2 are dominantly expressed in human and mouse heart tissues, and mutation in these genes lead to inherited cardiomyopathy that often involves arrhythmogenic cardiomyopathy (AC) (3–10). The complete deficiency of desmoglein-2, exhibited in homozygous knockout mice, is fatal during early embryogenesis, and *Dsg2*-depleted ES cells do not proliferate (11). Cardiac-specific *Dsg2* knockout mice are born with a functional heart but develop chamber dilatation with cardiomyocyte necrosis and interstitial fibrosis postnatally, modeling the human DCM phenotype (12,13). These data uniformly suggest that, in mice, the essential function of *DSG2* is to maintain the structural integrity of cardiac cells (11). In humans, heterozygous missense or nonsense mutations in *DSG2* have been identified among AC patients (5,14–16). Homozygous desmosome mutation is associated with a more severe phenotype, left ventricle (LV) involvement, higher sudden cardiac death risk and earlier onset of ventricular arrhythmias (17,18). Compound heterozygous mutations in *DSG2* (M1I/I333T) that lead to decreased desmoglein-2 expression cause severe juvenile-onset heart failure (19). To date, a homozygous stop-gain mutation in *DSG2* resulting from recessive inheritance has not been reported in a human case. Furthermore, the proof of concept to restore the deficient *DSG2* molecule in human cardiomyocytes has not been established.

Here, we identify a case of desmoglein-2-deficient cardiomyopathy caused by a rare homozygous stop-gain mutation. This patient exhibited uncontrollable ventricular arrhythmia, progressive biventricular dilatation and contractile dysfunction of the heart. Detailed histological examinations revealed disrupted desmosome structures and abnormal deposition of desmosome proteins in the patient’s myocardium. Cardiomyocytes differentiated from patient-derived induced pluripotent stem cells (iPSCs) recapitulated the structural fragility of the myocardium, and restoration of desmoglein-2 via homology-directed repair (HDR)-mediated genome editing corrected the pathological phenotypes. Furthermore, adeno-associated virus (AAV)-mediated replacement of the *DSG2* gene in differentiated cardiomyocytes increased the tissue strength in self-organized tissue rings (SOTRs) generated from R119X-induced pluripotent stem cell-derived cardiomyocytes (iPSC-CMs). Our findings indicate desmosome deficiency as the primary etiology of clinically diagnosed idiopathic DCM and provide the proof of concept of gene replacement therapy for cardiomyopathy.

## Results

### Identification of desmoglein-2-deficient cardiomyopathy in a patient with severe biventricular dilatation and contractile dysfunction

**Figure 1:**
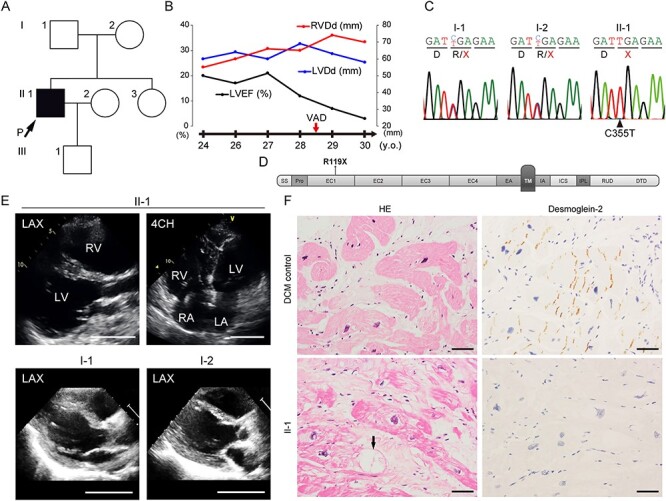
(**A**) Family pedigree of the patient. Only the proband indicated by the black square with the arrow was associated with DCM. (**B**) Clinical course and echocardiographic findings of the patient at ages 24–30 years. LVEF, left ventricular ejection fraction; LVDd, left ventricular diastolic diameter; RVDd, right ventricular diastolic diameter. (**C**) Result of direct Sanger sequence analysis using genomic DNA extracted from peripheral blood of the patient and his parents. Both parents (I-1 and I-2) were heterozygous for the C355T mutation, and the patient carried the homozygous C355T mutation in *DSG2*. Coding for the arginine residue (R) was changed to a stop codon (TGA). (**D**) Domain structure of desmoglein-2 (modified from Awad *et al*. (14)). SS, single peptide sequence; Pro, propeptide; EC, EC subdomains; EA, EC anchor; TM, transmembrane domain; IA, intracellular anchor; ICS, intracellular cadherin-typical sequence; IPL, intracellular proline-rich linker; RUD, repeated-unit domains; DTD, desmoglein-specific terminal domain. The R119X mutation is located in the EC1 domain. (**E**) Echocardiogram of the patient (II-1, 26 years old) and the parents (I-1, 61 years old and I-2, 55 years old). LAX, left parasternal long axis view; 4CH, four chamber view; RV, right ventricle; LA, left atrium; RA, right atrium. Scale bar: 50 mm. (**F**) Hematoxylin-eosin (HE) staining (left) and immunohistochemical analysis (right) of serial sections of the LV myocardium obtained from the DCM control (wild-type *DSG2*) (upper) or the patient (II-1) (lower) during VAD implantation. Cardiomyocytes show negative reactivity for desmoglein-2 in the patient (II-1). Black arrow indicates a vacuolated structure in a cardiomyocyte. Scale bar: 50 μm.

We encountered a patient with juvenile-onset sporadic idiopathic DCM who underwent ventricular assist device (VAD) implantation owing to severe cardiac dysfunction. The patient was normal at birth, with no apparent congenital cardiac disorder or somatic anomaly as far as we investigated from the available medical records and was without a familial history of cardiac disease (Fig. 1A). When the patient was 15 years old, abnormalities were initially found in an electrocardiogram, and further examination clarified a dilated heart, reduced left ventricular contraction and a high pulmonary arterial wedge pressure. The patient was finally diagnosed with idiopathic DCM and received standard medical therapies, including a β-blocker, to improve cardiac function (20,21). However, the LV ejection fraction gradually decreased, and echocardiography revealed biventricular dilatation (Fig. 1B). An electrocardiogram of the patient at age 21 shows intraventricular conduction disturbance and premature ventricular contraction (Supplementary Material, Fig. S1A). Atrial arrhythmias were not remarkable in the clinical course. Finally, the patient underwent emergent implantation of VAD at age 28 owing to recurrent and uncontrollable ventricular tachycardia. Genetic analysis using amplicon sequencing that targeted 404 cardiovascular genes identified a homozygous stop-gain mutation (c.C355T, p.R119X) in *DSG2* encoding desmoglein-2, which was inherited from his parents carrying heterozygous C355T mutation (Fig. 1C). Consanguineous relationship was not confirmed between the parents carrying R119X mutation as far as we investigated. Desmoglein-2 consists of N-terminal extracellular (EC) cadherin domains (EC1–EC4) that interact in *cis* and *trans* to form homo- and heteropolymers with desmoglein or desmocollin located in the intercellular space via the transmembrane domain and the C-terminal domain in the cytoplasm (3,5). The stop-gain mutation at residue R119 results in protein termination at the N-terminal EC1 domain (Fig. 1D). The C355T mutation in *DSG2* is an extremely rare variant in the ExAC database (22), with an allele frequency of 0.00002524 (1/65 454 in European population and 2/8512 in East Asian population), and none has been reported in the 1000 Genomes Project Consortium database (23), Exome Sequencing Project database (24,25) or Japanese Human Genetic Variation Database (26,27). This *DSG2* mutation was not seen among 7855 cardiomyopathy patients with DCM or arrhythmogenic right ventricular cardiomyopathy (ARVC) (28). The echocardiogram from our patient demonstrated significant dilatation of both ventricles (Fig. 1E and Supplementary Material, Fig. S1B). While both parents were heterozygous for R119X mutation in *DSG2,* neither exhibited morphological or functional abnormalities as evaluated by echocardiography (Fig. 1E and Supplementary Material, Fig. S1B) or repolarization abnormalities or arrhythmias as assessed by electrocardiography (Supplementary Material, Fig. S1A). Diffuse fibrosis and size variation of cardiomyocytes were observed in the patient’s left ventricular myocardium obtained during VAD implantation (Supplementary Material, Fig. S1C). Notably, significant abnormal intracellular vacuolated structures were observed in the patient’s myocardium as compared with the DCM control (Fig. 1F, arrow). Immunohistochemical analysis of heart sections clarified that immunoreactive signals of desmoglein-2 were not observed in intercalated disks, which is in contrast to that of the control (Fig. 1F).

### Loss of desmoglein-2 causes abnormal deposition of desmosome proteins and disruption of intercalated disks in cardiomyocytes

**Figure 2:**
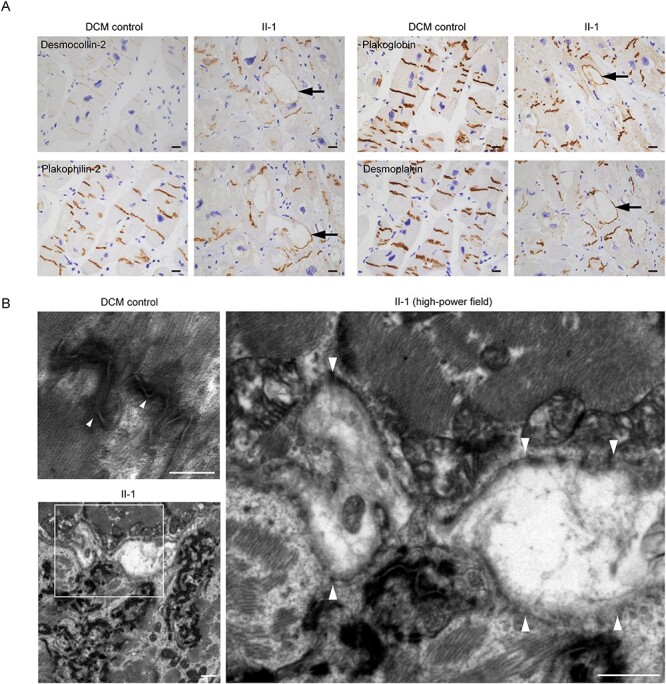
(**A**) Immunohistochemical analysis of the left ventricular myocardium obtained from the DCM control or the patient (II-1) using the indicated antibodies. Control samples were obtained from a DCM patient with advanced heart failure in whom pathological mutations of desmosome genes were not identified. Vacuolated structures are seen in cardiomyocytes of the patient (II-1) but not the DCM control. Desmosome proteins including desmocollin-2, plakophilin-2, plakoglobin and desmoplakin distributed in the rim of the vacuole in cardiomyocytes (black arrows). Scale bars: 20 μm. (B) Transmission electron microscope images of the LV myocardium obtained from DCM control (upper left) and the patient (II-1) (lower left). The white square in the lower left image is enlarged in the right image. Normal desmosome structures were observed in the DCM control (left upper, arrowheads). Disrupted desmosome (right, arrowheads) was observed. Scale bars: 500 nm (upper left image) and 1 μm (lower left and right images).

Desmocollin-2 interacts with desmoglein-2 and is localized in the EC space between adjacent cells (7). Armadillo protein family members (plakophilin-2 and plakoglobin) interact with plakin family members (desmoplakin) to form outer- and inner-dense plaque in desmosome structures (8,9). The distribution of these desmosome proteins in the patient’s myocardium was examined using serial sections with immunostaining. We observed severe disarray of the intercalated disks as compared with the DCM control and abnormal deposition of desmosome proteins in the cytoplasm and around the vacuolated structures (Fig. 2A). Transmission electron microscopy (TEM) analysis clarified that intercalated disk structures were severely disrupted in the patient’s myocardium compared with the DCM control (Fig. 2B). Strikingly, the intercalated disks were extensively disrupted (Fig. 2B, right).

### Generation of isogenic iPSC-CMs with restored desmoglein-2 expression by genome editing

**Figure 3:**
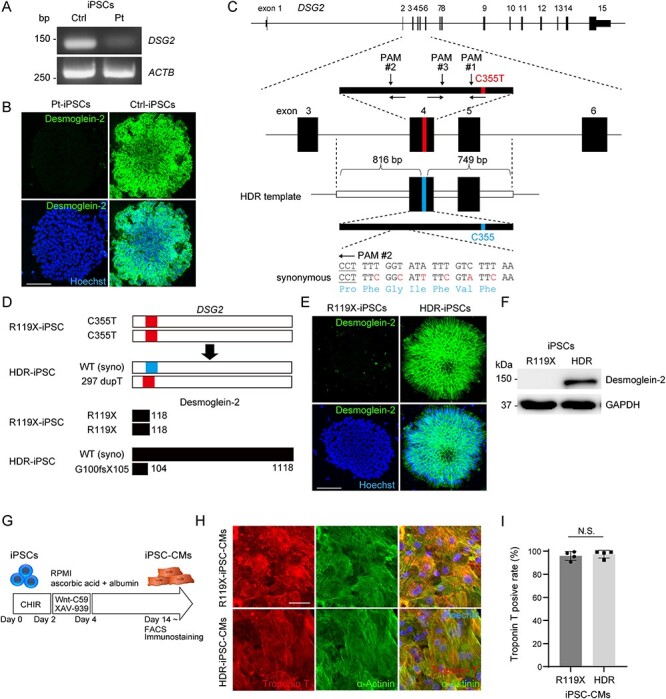
(**A**) cDNA was obtained from iPSCs generated from the control or patient carrying the *DSG2* R119X mutation, and the coding sequence of *DSG2* was amplified by PCR. Primer pairs targeting the coding sequence of *ACTB* were used for the control. (**B**) Pt- and Ctrl-iPSCs were fixed and immunostained with anti-desmoglein-2 antibody. Nuclei were detected by Hoechst staining. Scale bar: 100 μm. (**C**) The human *DSG2* gene consists of 15 exons (upper). Exons 3–6 are enlarged, and the targeted sites of genome editing around the C355T mutation at exon 4 are shown (middle). gRNA #1 specifically targets the allele containing C355T. gRNA #2 and #3 target a common sequence upstream of C355T. Design of the HDR repair template consisting of 816 bp 5′-terminal and 749 bp 3′-terminal homology arms corresponding to genomic sequences around exon 4 of *DSG2* (lower). Synonymous mutations were introduced to avoid Cas9-mediated re-cleavage. (**D**) Scheme of genomic sequence of *DSG2* (upper) and the translated amino-acid sequence of desmoglein-2 (lower) in R119X- and HDR-iPSCs. (**E**) R119X- and HDR-iPSCs were fixed and immunostained with anti-desmoglein-2 antibody. Scale bar: 100 μm. (**F**) Whole-cell lysates were extracted from R119X- and HDR-iPSCs and analyzed by western blotting using the indicated antibodies. (**G**) Time course for differentiation into cardiomyocytes (31). After differentiation, cells were analyzed by FACS and immunostaining. (**H**) R119X- and HDR-iPSC-CMs replated in 96-well plates on day 14 were fixed and immunostained on day 20 with the indicated antibodies. Scale bar: 50 μm. (**I**) Troponin T-positive ratio in FACS analysis of R119X-iPSC-CMs (*n* = 4) versus HDR-iPSC-CMs (*n* = 4) (95.9 ± 3.9 vs. 97.4 ± 3.3%, respectively; *P* = 0.665). Data were presented as the mean ± SD.

To generate a disease model of desmoglein-2-deficient cardiomyopathy, iPSCs were generated from peripheral blood mononuclear cells (PBMCs) obtained from the patient (Pt-iPSCs) and a control healthy donor subject (Ctrl-iPSCs). The Pt-iPSCs were positive for pluripotent markers (Supplementary Material, Fig. S2A) and carried a normal karyotype (Supplementary Material, Fig. S2B). mRNA expression of *DSG2* was significantly lower in Pt-iPSCs than in Ctrl-iPSCs (Fig. 3A). Immunostaining demonstrated that desmoglein-2 was completely absent in Pt-iPSCs (Fig. 3B). To precisely determine the functional consequence of desmoglein-2 deficiency in cardiomyocytes differentiated from iPSCs (iPSC-CMs), we sought to generate isogenic iPSCs with a repaired *DSG2* allele using clustered regularly interspaced short palindromic repeats (CRISPR)/CRISPR-associated protein 9 (Cas9) genome editing. First, we designed three gRNAs targeting three protospacer adjacent motif (PAM) sequences around the C355T mutation in exon 4 of *DSG2* (#1, #2 and #3) (Fig. 3C). gRNA #1 was designed to specifically target the mutated allele, including the C355T sequence evaluated by single-strand annealing assay (29,30) (Supplementary Material, Fig. S2C); however, this gRNA did not introduce non-homologous end joining (NHEJ) in iPSCs in repeated sessions of electroporation. gRNA #2 exhibited the highest cleavage activity (Supplementary Material, Fig. S2C) and efficiently induced NHEJ in Pt-iPSCs (Supplementary Material, Fig. S2D). Therefore, we chose gRNA #2 for further experiments. Next, we generated repair template DNA for HDR that contained the corrected C355 sequence flanked by 816 bp 5′- and 749 bp 3′-homology arms and introduced synonymous mutations into the upstream sequence of PAM#2 to avoid Cas9-mediated re-cleavage (Fig. 3C). The pX459 vector (30) encoding gRNA #2 and the generated repair template vector were transfected together into Pt-iPSCs, and the corrected clone was screened by Sanger sequencing and immunostaining. Finally, after repeated sessions of genome editing, we obtained an iPSC clone with heterozygously repaired alleles via HDR (HDR-iPSCs) (Fig. 3D and Supplementary Material, Fig. S2E). In HDR-iPSC, NHEJ was introduced into the allele encoding a frameshift mutation leading to a truncated desmoglein-2 protein with 104 amino acids. We also obtained an iPSC clone in which the stop-gain mutation in *DSG2* remained unchanged during the same selection procedure to use as a control for further experiments (R119X-iPSCs). The generated isogenic set of iPSCs were positive for pluripotent markers (Supplementary Material, Fig. S2A) and carried a normal karyotype (Supplementary Material, Fig. S2B). Immunostaining and western blot analysis demonstrated that the desmoglein-2 protein expression was recovered in HDR-iPSCs (Fig. 3E and F). The human iPSCs were differentiated into human iPSC-CMs by using a chemically defined protocol (31) (Fig. 3G), see Supplementary Material. Near day 10 after differentiation, both isogenic iPSC-CMs began to beat and exhibited no significant morphological differences (Fig. 3H). The differentiation efficiencies of the isogenic iPSC-CMs, as determined by fluorescence-activated cell sorting (FACS) analysis using anti-troponin T antibody, were >90% on day 14 after differentiation (Fig. 3I). The truncated protein product caused by R119X mutation was not detected in R119X-iPSC-CMs (Supplementary Material, Fig. S2F).

**Figure 4:**
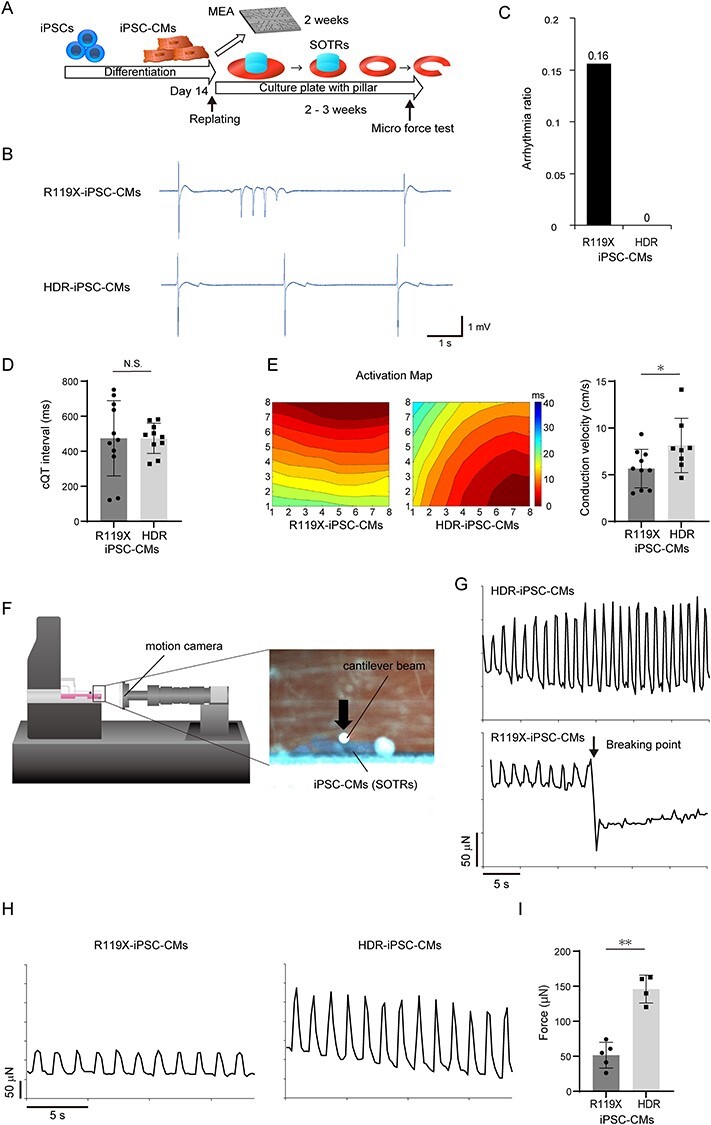
(**A**) Fourteen days after differentiation, both R119X- and HDR-iPSC-CMs were replated, and the MEA test was performed 2 weeks later. iPSC-CMs replated into 24-well plates with pillars at the center of the culture wells formed SOTRs within 2–3 weeks. SOTRs were linearized and analyzed by micro-force test. (**B**) The representative MEA records of R119X- and HDR-iPSC-CMs. (**C**) Arrhythmia ratio in R119X- and HDR-iPSC-CMs samples [R119X-iPSC-CMs (*n* = 5/32) vs. HDR-iPSC-CMs (*n* = 0/28), 0.16 vs. 0, respectively]. (**D**) Corrected QT intervals in R119X-iPSC-CMs (*n* = 11) and HDR-iPSC-CMs (*n* = 10) (474 ± 205 vs. 474 ± 82 ms, respectively; *P* = 0.995). Data were presented as the mean ± SD. (**E**) Representative activation maps (left) and conduction velocity (right) of R119X-iPSC-CMs (*n* = 10) and HDR-iPSC-CMs (*n* = 8) (5.7 ± 1.9 vs. 8.1 ± 2.7 cm/s, respectively; ^*^P < 0.05). Data were presented as the mean ± SD. (**F**) Linearized SOTRs were set on the flat surface stage then attached to the cantilever beam. The force was calculated from the cantilever beam deflection in response to differential displacement under increasing compression. Micro-motion of the cantilever beam and SOTR were detected by a motion camera located in front of the samples. (**G**) Representative micro-force traces in SOTRs generated from R119X-iPSC-CMs and HDR-iPSC-CMs. During increasing compression, the SOTR generated from R119X-iPSC-CMs suddenly broke. (**H**) Representative micro-force traces in SOTR generated from R119X-iPSC-CMs (left) and HDR-iPSC-CMs (right). (**I**) The average maximum forces were calculated from R119X-iPSC-CMs (*n* = 5) and HDR-iPSC-CMs (*n* = 4) (52 ± 16 vs. 145 ± 17 μN, respectively; ^*^^*^P < 0.01) and were reported as the average of 10 beats in each SOTR (mean ± SD).

### Desmoglein-2-deficient iPSC-CMs exhibited abnormal electrical activity, structural fragility and reduced force generation in SOTRs

The patient lacking desmoglein-2 expression in heart tissues manifested recurrent and uncontrollable ventricular arrhythmia. To investigate the functional consequence of desmoglein-2 deficiency in iPSC-CMs, electrical activity was measured using the multielectrode array (MEA) data acquisition system (32) (Fig. 4A). For quantitative evaluation, the occurrence of abnormal electrical activity in the isogenic iPSC-CMs was measured during a 1 min interval. As shown in Fig. 4B, abnormal ectopic electrical activities were observed in R119X-iPSC-CMs. Notably, the ectopic activities were not detected in HDR-iPSC-CMs in repeated experiments (Fig. 4B and C). Corrected QT interval represented by field potential duration (32) did not exhibit significant difference between R119X- and HDR-iPSC-CMs (Fig. 4D). Notably, conduction velocity calculated by activation maps obtained from MEA analysis was significantly decreased in R119X-iPSC-CMs compared with HDR-iPSC-CMs (Fig. 4E). Both ventricles of the patient with R119X mutation in *DSG2* were significantly enlarged, and the cardiac contractile function progressively decreased over the clinical course (Fig. 1B). We recently developed a simple device in modified culture plate on which iPSC-CMs form three-dimensional (3D) SOTRs that show higher maturation, including structural organization (33). To investigate the structural strength and force generation of the organized tissue lacking desmoglein-2 expression, we generated SOTRs from the isogenic iPSC-CMs and evaluated their contractile force using a MicroTester G2. Both isogenic iPSC-CMs were replated into 24-well plates with central pillars on day 14 after differentiation to form SOTRs (Fig. 4A). At 2–3 weeks after replating, SOTRs had formed in both R119X- and HDR-iPSC-CMs. The generated SOTRs were cut on one side, linearized, set on a flat surface stage and were attached by cantilever beam. The micro-force generated from the isogenic SOTRs was measured as the repulsive force of linearized SOTRs under increasing compression by cantilever beam (Fig. 4F). SOTRs generated from R119X-iPSC-CMs were easily broken during the compression process (*n* = 4/5 SOTRs) (Fig. 4G and Supplementary Material, Video S1), but SOTRs generated from HDR-iPSC-CMs did not break, and the force traces were continuously recorded during the measurement process (*n* = 0/4 SOTRs, Fig. 4G). The weak maximum force in SOTRs from R119X-iPSC-CMs was significantly recovered in SOTRs from HDR-iPSC-CMs (Fig. 4H and I and Supplementary Material, Videos S2 and S3). These data suggest that R119X-iPSC-CMs recapitulate the arrhythmogenicity and reduced cardiac contraction observed in the patient, and restoration of desmoglein-2 expression rescues these pathological phenotypes.

### Disturbed structural formation and disrupted desmosomes in desmoglein-2-deficient iPSC-CMs

**Figure 5:**
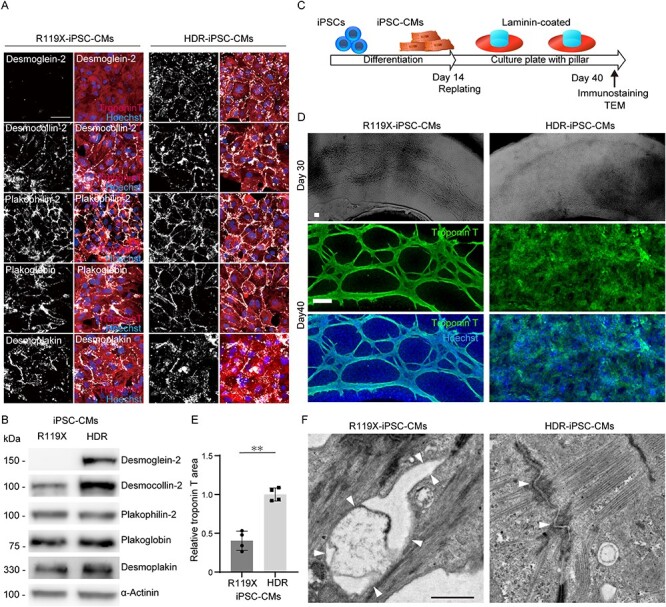
(**A**) R119X- and HDR-iPSC-CMs replated in 96-well plates on day 14 were fixed and immunostained on day 20 with the indicated antibodies. Scale bar: 50 μm. (**B**) Whole-cell lysates were extracted from R119X- and HDR-iPSC-CMs on day 14 and analyzed by western blotting using the indicated antibodies. (**C**) Fourteen days after differentiation, both R119X- and HDR-iPSC-CMs were replated into 24-well plates with pillars precoated with laminin for continuous observation of the 2D structure. Replated iPSC-CMs were observed over time and then analyzed by immunostaining and TEM. (**D**) Bright field images on day 32 (upper) and immunostaining images after fixation on day 40 (middle and lower) of R119X- and HDR-iPSC-CMs replated in 24-well plates with pillars. Scale bars: 100 μm. (**E**) Troponin-T-positive area normalized by Hoechst area either in R119X- and HDR-iPSC-CMs was calculated (50) using Image J software (National Institutes of Health, Bethesda, MD, USA). The relative value of R119X-iPSC-CMs normalized to that of HDR-iPSC-CMs is shown (0.40 ± 0.12 vs. 1.0 ± 0.08, respectively; ^*^^*^P < 0.001; *n* = 4). Data were presented as the mean ± SD. (**F**) Transmission electron microscope images of the desmosome obtained from R119X- and HDR- iPSC-CMs on day 40. The terminated desmosomes and disrupted intercalated disk (arrowheads) were observed in R119X-iPSC-CMs. The normal desmosome structures (arrowheads) were observed in HDR-iPSC-CMs. Scale bar: 1 μm.

To reveal the downstream effects of desmoglein-2 deficiency, the expression and localization of desmosome proteins were determined by immunostaining and western blot analysis. Normal desmoglein-2 expression was recovered in HDR-iPSC-CMs and was localized at the cell–cell periphery with a dotted distribution (Fig. 5A and B). Interestingly, the protein expression level of desmocollin-2, a desmosome cadherin that forms homo- and hetero-dimers with desmoglein-2 (34), was significantly decreased in R119X-iPSC-CMs, but no significant difference in other desmosome proteins (plakophilin-2, plakoglobin or desmoplakin) was observed between isogenic iPSC-CMs (Fig. 5A and B and Supplementary Material, Fig. S3A). The expression levels of N-cadherin or connexin 43 were not significantly affected in R119X-iPSC-CMs (Supplementary Material, Fig. S3B). To reveal morphological changes in R119X-iPSC-CMs during the formation of SOTRs, the isogenic iPSC-CMs were replated onto laminin-coated 24-well plates with pillars for continuous observation at the two-dimensional (2D) level. The iPSC-CMs were observed over time and fixed on day 40 for immunostaining and TEM (Fig. 5C). These analyses revealed that the cardiomyocytes and a subset of non-cardiomyocytes gradually formed connecting structures. One to two weeks after replating, hole-like structures appeared in the R119X-iPSC-CMs, which increased in area gradually over time (Fig. 5D and Supplementary Material, Fig. S3C). Immunostaining experiments using anti-troponin T antibody clarified that R119X-iPSC-CMs formed a spiderweb-like fiber structure; these morphological abnormalities were significantly recovered in HDR-iPSC-CMs (Fig. 5D and E). TEM demonstrated that the desmosome structures were disrupted in R119X-iPSC-CMs but were restored in HDR-iPSC-CMs (Fig. 5F). These findings suggest that desmoglein-2 deficiency does not significantly affect the proteins localized at the outer- or inner-dense plaques (9) but decreases the amount of membrane-bound co-localized desmocollin-2 and that loss of both desmosome cadherins severely disrupts the adhering process and structural formation in iPSC-CMs.

### A‌AV-mediated *DSG2* gene replacement recovered the maximum force of SOTRs in R119X-iPSC-CMs

**Figure 6:**
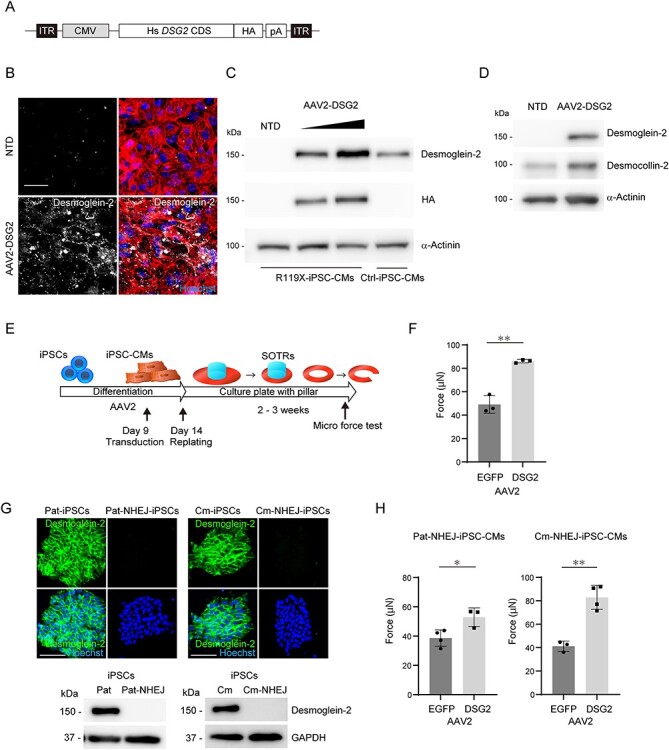
(**A**) The 3′-terminally HA-tagged full length human *DSG2* coding sequence (Hs *DSG2* CDS) was subcloned into an expression vector with a CMV promoter and poly A sequence (pA) to generate AAV2. ITR, inverted terminal repeats. (**B**) Four days after replating, R119X-iPSC-CMs in 96-well plates were transduced with 2.0 × 10^4^ vg/cell of AAV2-DSG2. Seven days after transduction, cells were fixed and immunostained with the indicated antibodies. NTD, non-transduction control. Scale bar: 50 μm. (**C**) On day 21 after differentiation, R119X-iPSC-CMs were transduced with 2.0 × 10^4^ or 6.0 × 10^4^ vg/cell of AAV2-DSG2. Seven days after transduction, whole-cell lysates were extracted and analyzed by western blotting using the indicated antibodies. Whole-cell lysates extracted from Ctrl-iPSC-CMs were used as the control. (**D**) On day 9 after differentiation, R119X-iPSC-CMs were transduced with 2.0 × 10^4^ vg/cell of AAV2-DSG2 and then replated into 24-well plates with pillars on day 14. Whole-cell lysates were prepared on day 30 and analyzed by western blotting using the indicated antibodies. (**E**) On day 9 after differentiation, R119X-iPSC-CMs were transduced with 2.0 × 10^4^ vg/cell of AAV2-DSG2 or AAV2-EGFP. Both types of cardiomyocytes were replated on day 14 to generate SOTRs, and the micro-force test was performed on the generated SOTRs. (**F**) The average maximum forces were calculated from SOTRs transduced with AAV2-EGFP (*n* = 3) and with AAV2-DSG2 (*n* = 3) (49 ± 6 vs. 86 ± 1 μN, respectively; ^*^^*^P < 0.01) and were reported as the average of 10 beats in each SOTR (mean ± SD). (**G**) Pat-, Pat-NHEJ-, Cm- and Cm-NHEJ-iPSCs were fixed and immunostained with anti-desmoglein-2 antibody. Nuclei were detected by Hoechst staining. Scale bar: 100 μm. Whole-cell lysates were extracted from Pat-, Pat-NHEJ-, Cm- and Cm-NHEJ-iPSCs and analyzed by western blotting using the indicated antibodies. (**H**) On day 9 after differentiation, Pat-NHEJ-iPSC-CMs or Cm-NHEJ-iPSC-CMs were transduced with 2.0 × 10^4^ vg/cell of AAV2-DSG2 or AAV2-EGFP. Both types of cardiomyocytes were replated on day 14 to generate SOTRs, and the micro-force test was performed on the generated SOTRs. The average maximum forces were calculated from Pat-NHEJ-iPSC-CMs transduced with AAV2-EGFP (*n* = 4) or AAV2-DSG2 (*n* = 3) (39 ± 5 vs. 53 ± 5 μN, respectively; ^*^P < 0.05) and Cm-NHEJ-iPSC-CMs transduced with AAV2-EGFP (*n* = 3) or AAV2-DSG2 (*n* = 4) (41 ± 4 vs. 83 ± 9 μN, respectively; ^*^^*^P < 0.01) and were reported as the average of 10 beats in each SOTR (mean ± SD).

R119X-iPSC-CMs differentiated normally and initially showed no significant abnormalities but demonstrated structural fragility after SOTR formation. These findings prompted us to evaluate whether replacing the *DSG2* gene after differentiation prevented the pathological phenotype observed in R119X-iPSC-CMs. We generated AAV-2 encoding a 3′-terminally hemagglutinin (HA)-tagged full-length human *DSG2* gene driven by the CMV promoter (AAV2-DSG2, Fig. 6A) because AAV2 transduces human iPSC-CMs with high efficiency in our experimental conditions (35) (Supplementary Material, Fig. S4A). Overexpressed desmoglein-2 was localized at the cell–cell periphery in R119X-iPSC-CMs (Fig. 6B). Transduction of these cells with AAV2-DSG2 at 2.0 × 10^4^ vg/cell restored desmoglein-2 expression at a level comparable with that of the endogenous gene in Ctrl-iPSC-CMs (Fig. 6C). Protein expression levels of desmocollin-2 were recovered in R119X-iPSC-CMs transduced with AAV2-DSG2 (Fig. 6D). R119X-iPSC-CMs were transduced with AAV2-DSG2 or AAV2-enhanced green fluorescent protein (EGFP) as a control on day 9 after differentiation, then replated into 24-well plates with central pillars on day 14 to induce SOTR formation (Fig. 6E). Micro-force tests of SOTRs showed that AAV2-mediated *DSG2* gene replacement significantly recovered the maximum force in R119X-iPSC-CMs (Fig. 6F).

### NHEJ-mediated introduction of protein-truncating mutations in isogenic iPSC-CMs and functional rescue using AAV-mediated *DSG2* gene replacement

Our experimental findings using isogenic iPSC-CMs demonstrated that HDR-mediated correction of stop-gain mutation significantly recovered the decreased maximum force in R119X-iPSC-CMs. To further confirm the pathological relevance of desmoglein-2 deficiency, we introduced NHEJ-mediated frameshift mutation into additional two iPSCs generated from the father of the patient (I-1, Fig. 1A) carrying heterozygous R119X mutation [named Paternal-iPSC (Pat-iPSC)] and from the other cardiomyopathy patient in which the pathogenic variant was not identified in *DSG2* [named cardiomyopathy-iPSC (Cm-iPSC)]. NHEJ-mediated homozygous frameshift mutations were introduced into these isogenic iPSCs (Pat-NHEJ-iPSC and Cm-NHEJ-iPSC), and desmoglein-2 protein was terminated within 110 amino acids (Supplementary Material, Fig. S4B and C). Desmoglein-2 expression was abolished both in immunostaining and western blot analysis (Fig. 6G). Desmoglein-2 deficiency in these isogenic iPSC-CMs did not significantly affect the distribution of desmocollin-2, plakophilin-2, plakoglobin or desmoplakin at cell borders but decreased the protein expression levels of desmocollin-2 (Supplementary Material, Fig. S5A and B). Importantly, AAV-mediated *DSG2* gene replacement significantly recovered the maximum force in the both SOTRs generated from Pat-NHEJ-iPSC-CMs and Cm-NHEJ-iPSC-CMs carrying the introduced mutations (Fig. 6H).

## Discussion

In this report, we identified a case of desmoglein-2-deficient cardiomyopathy caused by a homozygous stop-gain mutation, which was initially diagnosed as idiopathic DCM. Homozygous missense mutation (36) and a compound heterozygous mutation composed of missense and nonsense mutations in severe ARVC cases (14) have been previously reported. However, to the best of our knowledge goes, no case involving a homozygous *DSG2* nonsense mutation leading to null protein expression has been reported in humans. Although mice deficient in *Dsg2* are embryonic lethal and *Dsg2*-depleted ES cells do not proliferate (11), the human patient completely lacking desmoglein-2 expression was normal at birth, with no apparent anomaly detectable by ordinary medical examinations. These findings and the subsequent clinical course of the patient suggest that the desmoglein-2-deficient human heart is compensated during the embryonic stage but cannot tolerate the continuous hemodynamic load after birth. Histopathological analysis via immunohistochemistry and TEM revealed striking features in the patient’s left ventricular myocardium, including abnormal deposition of desmosome proteins at the periphery of vacuolated structures and disrupted intercalated disks. Cytoplasmic vacuole formation is reported in the myocardium of an ARVC patient with a heterozygous frameshift mutation in *DSG2* (15). Genetic mutations in cadherin proteins, including desmogleins, can interfere with their assembly into membranes and internalization into the cytoplasm (37,38). The continuous hemodynamic load on cell-to-cell junctions lacking desmoglein-2 further exacerbates the disruption of desmosomes and may promote abnormal turnover of desmosome proteins. TEM analyses clarify that gaps in desmosomes are wider in ARVC patients (15,39) and desmosomes are internalized into the cytosol after wound formation in mouse skin (40). The disrupted intercalated disks and abnormal deposition of desmosome proteins at vacuolated structures observed in the patient’s myocardium exemplify the excessive tissue injury owing to structural fragility caused by desmoglein-2 deficiency.

Many heterozygous missense, nonsense and frameshift mutations in *DSG2* have been identified in patients with ARVC (5). Patients with a heterozygous frameshift mutation (E418fsX419 or G678fsX681) or heterozygous stop-gain mutation (Q558X) were diagnosed with ARVC based on task force criteria (15). Unexpectedly, both parents of the patient harboring heterozygous R119X mutation showed neither symptoms nor abnormalities in electrocardiograms or in echocardiograms, suggesting that the haploinsufficiency caused by R119X mutation does not significantly affect cardiac function, at least in the case of this family. These phenotype–genotype correlations are important findings because they suggest that at least half-dose restoration of desmoglein-2 expression may rescue the pathological phenotype caused by the homozygous R119X mutation. The possibility of such rescue is consistent with our experimental findings that abnormal electrical activity, structural fragility, reduced contraction force and disturbed myocardial fiber formation in R119X-iPSC-CMs were rescued in HDR-iPSC-CMs carrying a heterozygously repaired allele.

Surprisingly, desmoglein-2 deficiency in iPSC-CMs did not significantly affect the expression or localization of plakophilin-2, plakoglobin or desmoplakin, which are the major components of the outer dense plaque (8,41). The immunoreactive signals of plakoglobin are significantly depressed in myocardium in ARVC cases with genetic mutations in desmosome genes (42,43). On the other hand, cardiomyocyte-specific knockout of *Dsg2* do not significantly affect the expression or localization of plakophilin-2, plakoglobin or desmoplakin in myocardium in 12-week-old mice (12). From these previous findings and from our experimental data using iPSC-CMs, we speculate that the downstream effects owing to desmoglein-2 deficiency may vary depending on species or disease models and desmoglein-2 deficiency alone may not significantly affect the outer plaque proteins at least in human iPSC-CMs under our experimental conditions. On the other hand, desmoglein-2 deficiency significantly decreased the expression of desmocollin-2 in iPSC-CMs, suggesting that disturbed hetero-dimer formation may affect the stability of desmocollin-2. A previous study reports that a patient carrying a homozygous deletion of *PKP2* encoding plakophilin-2, a molecular scaffold protein in the outer dense plaque, manifested fetal cardiac failure and died at the age of 12 days (44). The relatively lower effect of desmoglein-2 deficiency on other desmosome proteins may account for the later appearance of the clinical phenotype of this patient, more than 10 years after birth. Although our established isogenic iPSC-CMs recapitulate the structural fragility and reduced contractile function of the patient, the striking histopathological features observed in the patient’s myocardium, including vacuolated structures and abnormal deposition of desmosome proteins, were not detected in the iPSC-CMs after differentiation. Full recapitulation of the detailed molecular phenotypes caused by desmoglein-2 deficiency will require technological improvements to study cell maturation and organoid formation using the isogenic iPSC-CMs.

The structural fragility caused by desmoglein-2 deficiency accounts for the observed clinical course of our patient, including juvenile onset, rapidly progressive biventricular dilatation and no improvement with β-blocker therapy. Increased arrhythmia ratio, disturbed conduction velocity evaluated by MEA analysis and abnormal spiderweb-like fiber structure observed in R119X-iPSC-CMs may account for the clinically detected intraventricular conduction disturbance and uncontrollable lethal ventricular arrhythmia in this patient. Contractile dysfunction in LV (ARVC with LV involvement) is found in 76% of ARVC cases (45) and is associated with arrhythmic events, more severe cardiomegaly, inflammatory infiltrates and heart failure (45,46). Of the known desmosome genes identified in ARVC with LV involvement (*DSG2*, *DSC2*, *DSP*, *PLN* and *TMEM43*) (17,18,47), the *DSG2* mutations show the highest prevalence, with a frequency of 20–50% (17,18). Specifically, a compound heterozygous mutation in *DSG2* (p.M1I/p.I333T) results in near-abolishment of myocardial desmoglein-2 expression and leads to severe biventricular heart failure (19). Recently, F531C mutation in *DSG2* has been identified as a homozygous founder variant that causes biventricular heart failure with 100% penetrance in East Asia (48). Of note, R119X mutation has been identified in two cases in the East Asian population in the ExAC database (22). These epidemiologic data combined with our findings suggest that desmoglein-2 deficiency might be concealed among the genetically undiagnosed idiopathic DCM patients with severe heart failure. AAV2-mediated gene replacement of *DSG2* after differentiation recovered the SOTR contraction force generated by R119X-iPSC-CMs lacking desmoglein-2 expression. These data suggest that gene replacement therapy to restore desmoglein-2 before disease progression could be an effective upstream therapy, potentially preventing the transition to advanced heart failure. No treatment other than heart transplantation is available for terminal-stage heart failure patients. Thus, another therapeutic approach based on the underlying molecular mechanism of inherited cardiomyopathy is urgently needed. Our findings using patient-derived isogenic disease model cells provide the conceptual basis for developing a novel therapeutic strategy.

## Materials and Methods

See Supplementary Methods for further details.

### Human sample

The use of human samples and the genomic analysis were approved by the Ethics Committee of Osaka University Hospital, and written informed consent was obtained. This investigation conforms to Ethical Guidelines for Medical and Health Research Involving Human Subjects in Japan and to all principles outlined by the Declaration of Helsinki.

### Generation of human iPSC clones

Patient-derived iPSC and healthy control iPSC clones were generated from PBMCs. Briefly, PBMCs were separated from peripheral whole blood using Ficoll-Plaque (GE). Reprogramming was performed by Sendai virus vectors with OCT3/4, SOX2, KLF4 and c-MYC (CytoTune-iPS 2.0 Sendai Reprogramming Kit, Life Technologies). Twenty-four hours after infection, PBMCs were seeded on laminin-coated plate (iMatrix-511, MATRIXOME, Osaka, Japan). iPS cells were maintained on laminin-coated plate with medium (StemFit AK02, AJINOMOTO) (49).

### Generation of SOTRs

The 3D tissue was fabricated according to the previous report (33). Before cell seeding, single CMs were filtered using a 40 μm cell strainer (BD Falcon; Becton Dickenson, Franklin Lakes, NJ, USA) and were resuspended at a density of 2 × 10^6^ cells/ml in serum-supplemented cardiac differentiation culture medium containing 40% high glucose DMEM (Sigma-Aldrich), 40% IMDM (Sigma-Aldrich), 20% fetal bovine serum (FBS; Gibco, USA), 1% minimum essential medium non-essential amino acid solution (Sigma-Aldrich) and 0.1% penicillin–streptomycin (Gibco, USA), 0.5% L-glutamine (Sigma-Aldrich). There were 4 × 10^5^ cells plated in each PDMS (SYLGARD 184; Dow Corning, Midland, MI, USA) culture well with a 3 mm pillar. After plating, CMs settled in the wells, aggregated and congregated around the central pillar to form densely packed tissue rings within 7 days. The medium was changed to serum-free medium from day 2. After that, fresh medium was changed every 4 days.

### Force test using MicroTester G2

The CM contractility of iPSC-CMs was measured using a micron-scale mechanical testing system (MicroTester G2; CellScale Biomaterials Testing, Waterloo, ON, Canada). The 3D tissue was removed from the pillar and was cut into a strip that was fixed onto a stage and immersed in 37°C culture medium. A cantilever beam (diameter, 0.30 mm) was pressed onto the 3D tissue. During force recording, the beam was held, and the force was calculated by cantilever beam deflection in response to differential displacement under increasing compression.

### Statistical analysis

Unless otherwise noted, data are presented as the mean ± SD from at least three independent experiments. Unpaired *t*-test (for two-sample analyses only) was performed for statistical analysis. For more than three samples, ANOVA followed by Tukey’s HSD test was performed. *P* < 0.05 was considered statistically significant, and all statistical analyses were performed using JMP version 14.3.0 (SAS Institute Inc., Tokyo, JAPAN).

## Supplementary Material

Supplementary_Figure_HMG-2021-CE-00080_Shiba_ddab127

Supplementary_table_1_HMG-2021-CE-00080_Shiba_ddab127

Supplementary_table_2_HMG-2021-CE-00080_Shiba_ddab127

Supplementary_video_1_HMG-2021-CE-00080_Shiba_ddab127

Supplementary_video_2_HMG-2021-CE-00080_Shiba_ddab127

Supplementary_video_3_HMG-2021-CE-00080_Shiba_ddab127

Supplementary_material_HMG-2021-CE-00080_Shiba_ddab127
