# Supplementary material for: Phenotypic recapitulation and correction of desmoglein-2-deficient cardiomyopathy using human-induced pluripotent stem cell-derived cardiomyocytes
Source: Hum Mol Genet. 2021 May 5;30(15):1384–97. doi: 10.1093/hmg/ddab127 (PMC8283207; doi:10.1093/hmg/ddab127)
Supplement: Supplementary_Figure_HMG-2021-CE-00080_Shiba_ddab127 [file supplementary_figure_hmg-2021-ce-00080_shiba_ddab127.pdf]

A

II-1 I-1 I-2

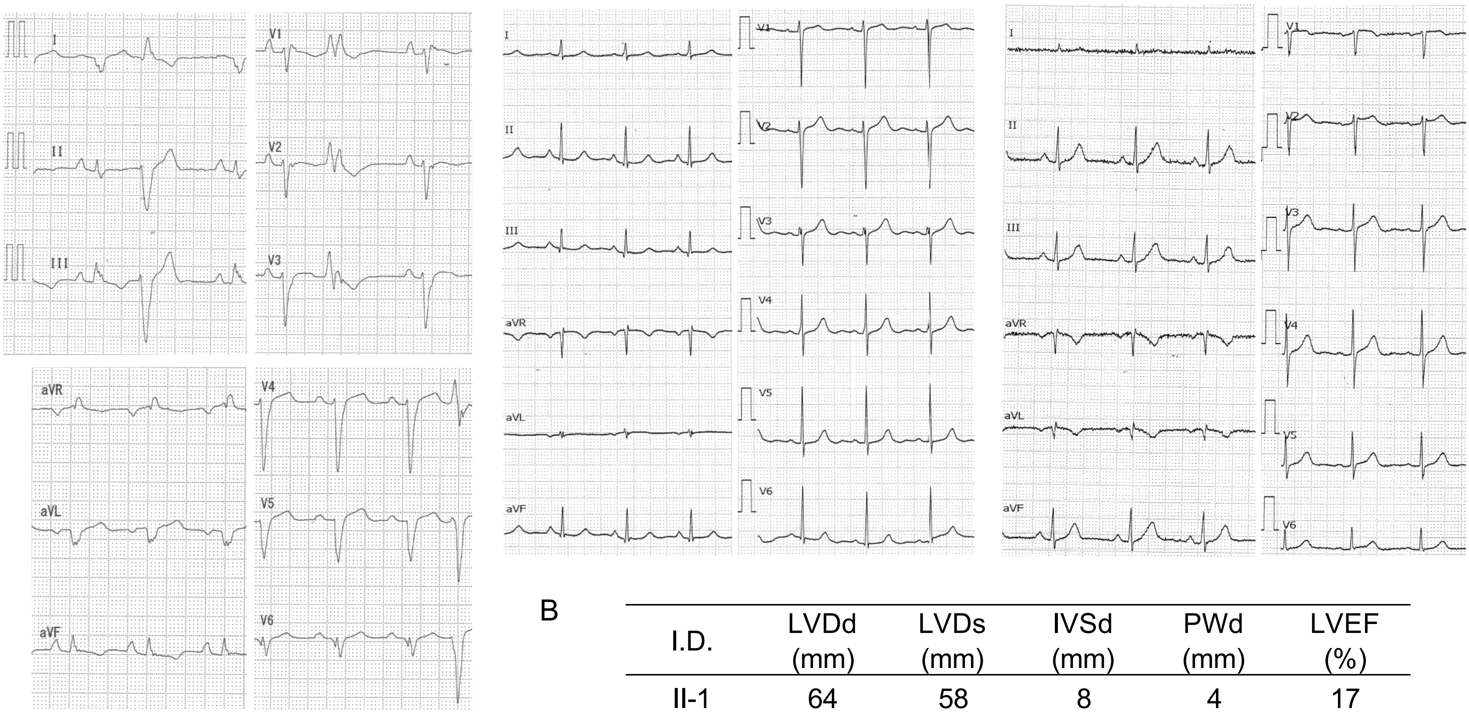

B

I.D.	LVDd (mm)	LVDs (mm)	IVSd (mm)	PWd (mm)	LVEF (%)
II-1	64	58	8	4	17
I-1	48	28	7	8	67
I-2	42	25	7	7	66

C

II-1

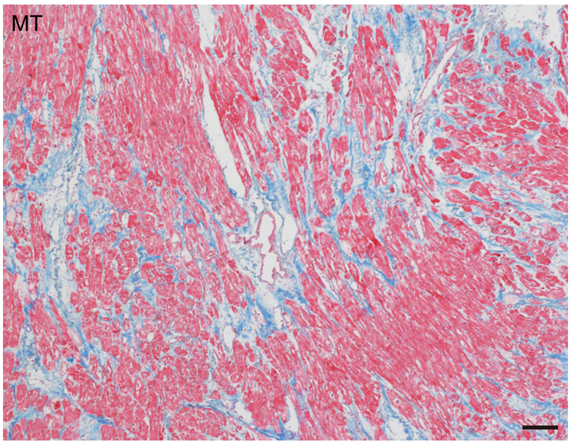

Supplementary_Figure_2

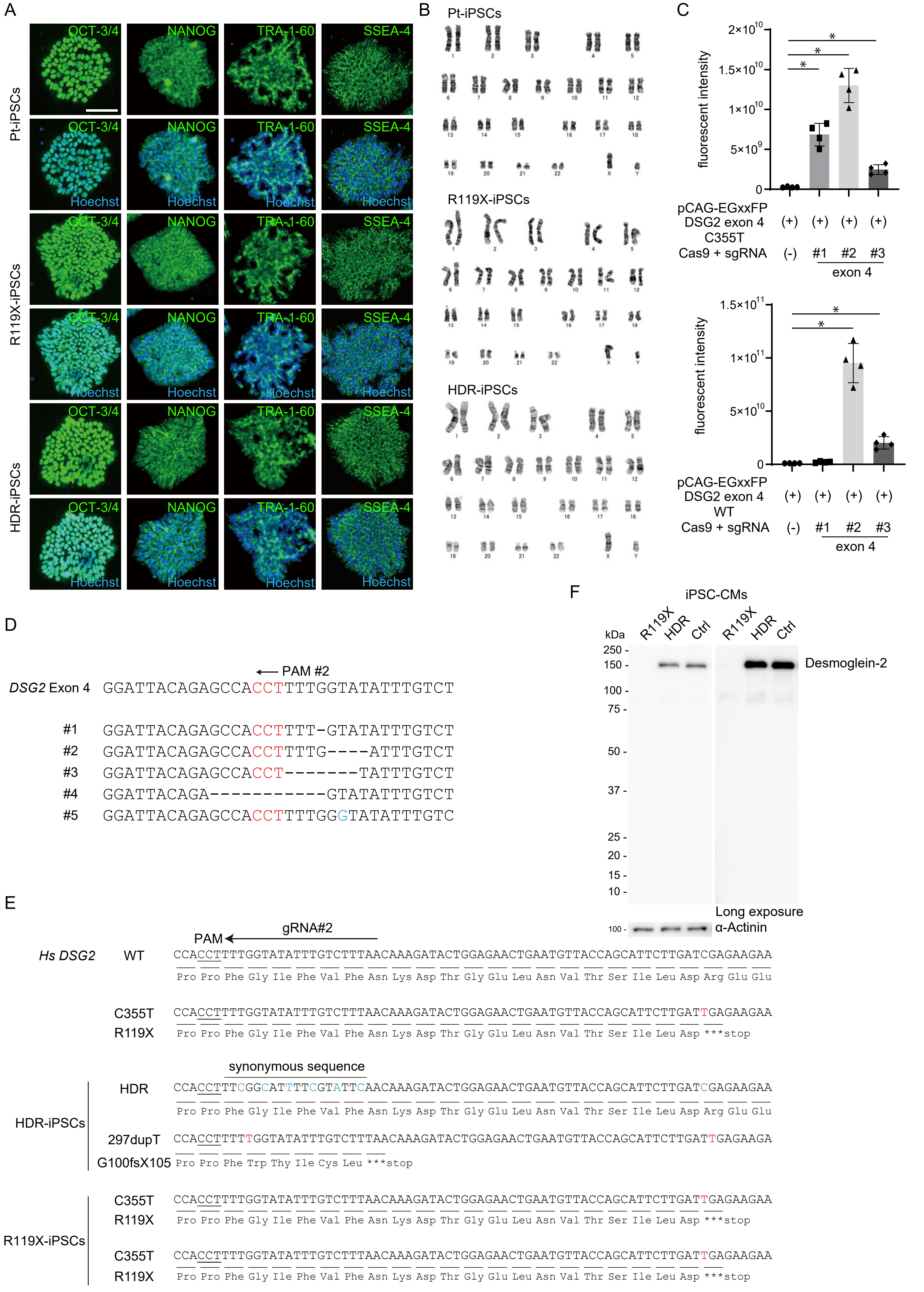

Supplementary_Figure_3

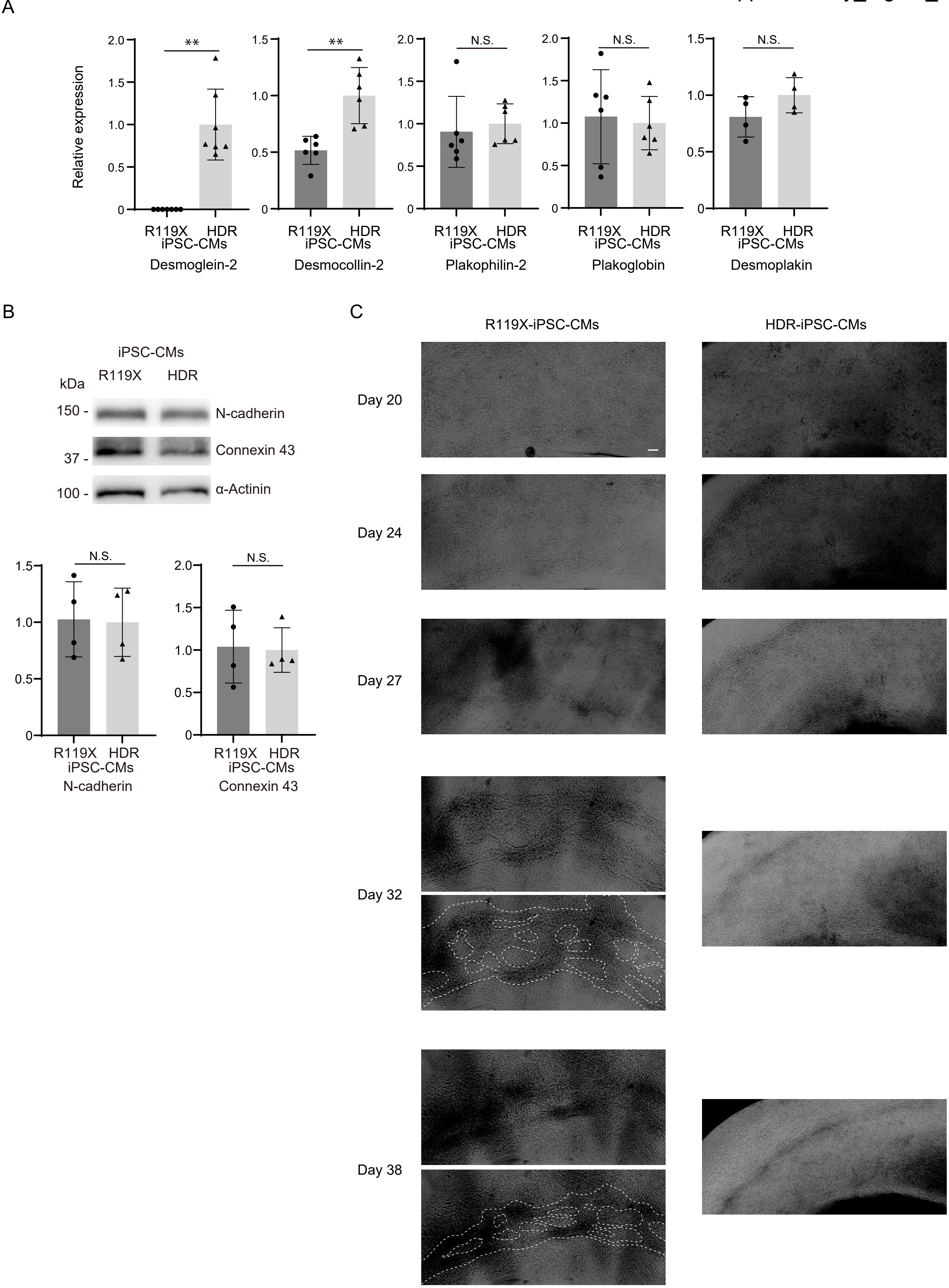

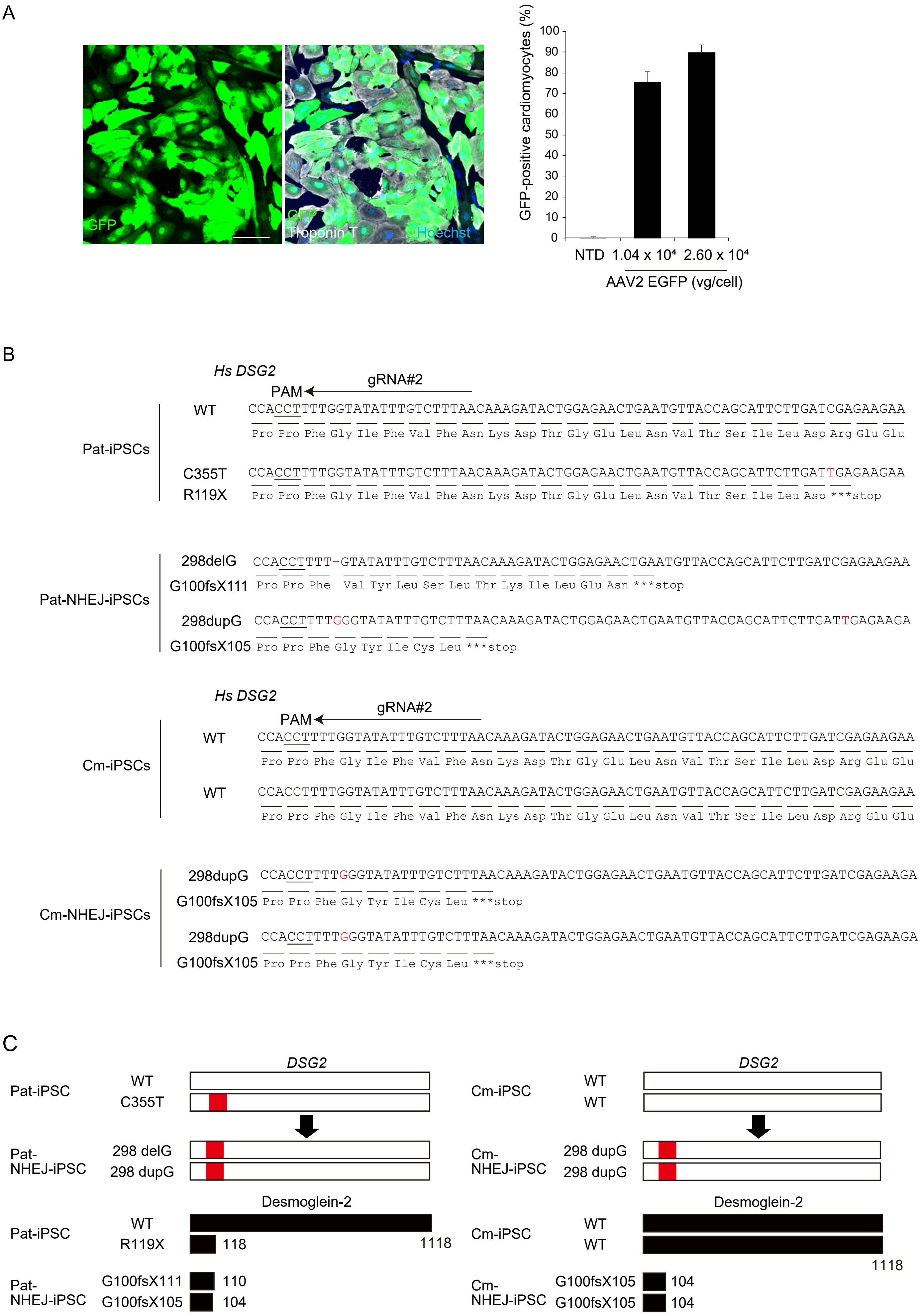
A
GFP
GFP
Troponin T
Hoechst
GFP-positive cardiomyocytes (%)
0
10
20
30
40
50
60
70
80
90
100
NTD
1.04 x 10⁴
2.60 x 10⁴
AAV2 EGFP (vg/cell)
B
Hs DSG2
gRNA#2
PAM
Pat-iPSCs
WT
CCACCTTTTGGTATATTTGTCTTTAACAAAGATACTGGAGAACTGAATGTTACCAGCATTCTTGATCGAGAAGAA
Pro Pro Phe Gly Ile Phe Val Phe Asn Lys Asp Thr Gly Glu Leu Asn Val Thr Ser Ile Leu Asp Arg Glu Glu
C355T
R119X
CCACCTTTTGGTATATTTGTCTTTAACAAAGATACTGGAGAACTGAATGTTACCAGCATTCTTGATTGAGAAGAA
Pro Pro Phe Gly Ile Phe Val Phe Asn Lys Asp Thr Gly Glu Leu Asn Val Thr Ser Ile Leu Asp ***stop
Pat-NHEJ-iPSCs
298delG
G100fsX111
CCACCTTTT-GTATATTTGTCTTTAACAAAGATACTGGAGAACTGAATGTTACCAGCATTCTTGATCGAGAAGAA
Pro Pro Phe Val Tyr Leu Ser Leu Thr Lys Ile Leu Glu Asn ***stop
298dupG
G100fsX105
CCACCTTTTGGGTATATTTGTCTTTAACAAAGATACTGGAGAACTGAATGTTACCAGCATTCTTGATTGAGAAGA
Pro Pro Phe Gly Tyr Ile Cys Leu ***stop
Hs DSG2
gRNA#2
PAM
Cm-iPSCs
WT
CCACCTTTTGGTATATTTGTCTTTAACAAAGATACTGGAGAACTGAATGTTACCAGCATTCTTGATCGAGAAGAA
Pro Pro Phe Gly Ile Phe Val Phe Asn Lys Asp Thr Gly Glu Leu Asn Val Thr Ser Ile Leu Asp Arg Glu Glu
WT
CCACCTTTTGGTATATTTGTCTTTAACAAAGATACTGGAGAACTGAATGTTACCAGCATTCTTGATCGAGAAGAA
Pro Pro Phe Gly Ile Phe Val Phe Asn Lys Asp Thr Gly Glu Leu Asn Val Thr Ser Ile Leu Asp Arg Glu Glu
Cm-NHEJ-iPSCs
298dupG
G100fsX105
CCACCTTTTGGGTATATTTGTCTTTAACAAAGATACTGGAGAACTGAATGTTACCAGCATTCTTGATCGAGAAGA
Pro Pro Phe Gly Tyr Ile Cys Leu ***stop
298dupG
G100fsX105
CCACCTTTTGGGTATATTTGTCTTTAACAAAGATACTGGAGAACTGAATGTTACCAGCATTCTTGATCGAGAAGA
Pro Pro Phe Gly Tyr Ile Cys Leu ***stop
C
DSG2
Pat-iPSC
WT
C355T
Pat-NHEJ-iPSC
298 delG
298 dupG
Desmoglein-2
Pat-iPSC
WT
R119X
118
1118
Pat-NHEJ-iPSC
G100fsX111
110
G100fsX105
104
DSG2
Cm-iPSC
WT
WT
Cm-NHEJ-iPSC
298 dupG
298 dupG
Desmoglein-2
Cm-iPSC
WT
WT
1118
Cm-NHEJ-iPSC
G100fsX105
104
G100fsX105
104

A

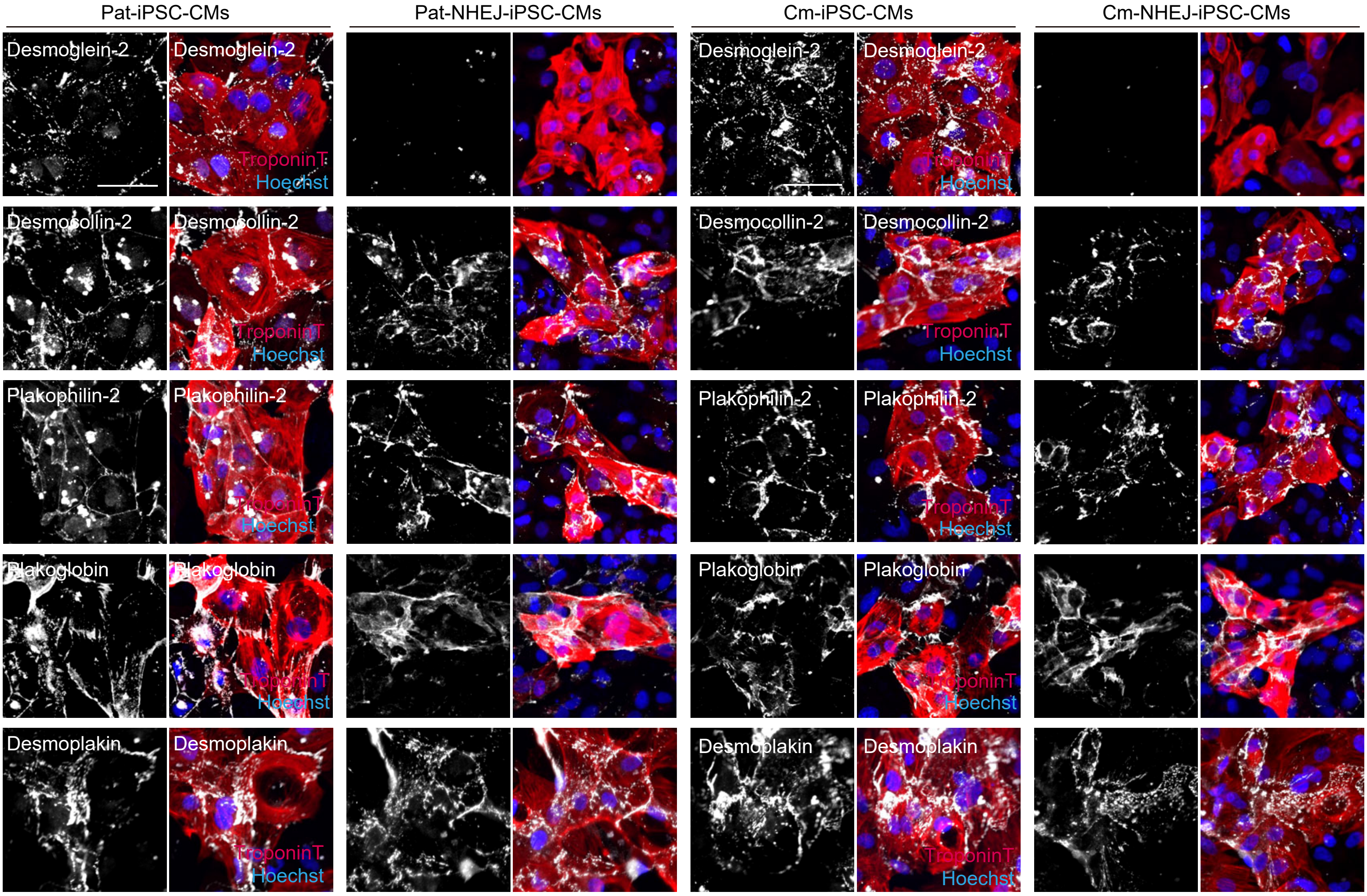
Pat-iPSC-CMs
Pat-NHEJ-iPSC-CMs
Cm-iPSC-CMs
Cm-NHEJ-iPSC-CMs
Desmoglein-2
TroponinT
Hoechst
Desmocollin-2
Plakophilin-2
Plakoglobin
Desmoplakin

B

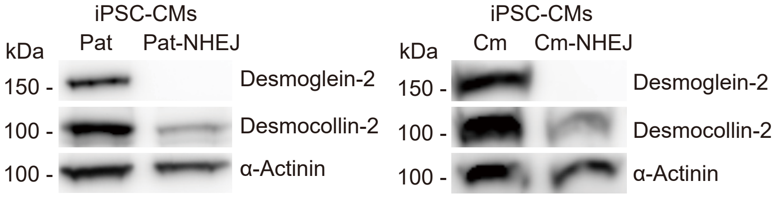
iPSC-CMs
kDa
Pat
Pat-NHEJ
150 -
100 -
100 -
Desmoglein-2
Desmocollin-2
α-Actinin
iPSC-CMs
kDa
Cm
Cm-NHEJ
150 -
100 -
100 -
Desmoglein-2
Desmocollin-2
α-Actinin
