# Supplementary material for: Phenotypic recapitulation and correction of desmoglein-2-deficient cardiomyopathy using human-induced pluripotent stem cell-derived cardiomyocytes
Source: Hum Mol Genet. 2021 May 5;30(15):1384–97. doi: 10.1093/hmg/ddab127 (PMC8283207; doi:10.1093/hmg/ddab127)
Supplement: Supplementary_material_HMG-2021-CE-00080_Shiba_ddab127 [file supplementary_material_hmg-2021-ce-00080_shiba_ddab127.docx]

**Supplementary Materials**

**Supplementary Methods**

***Immunohistochemical Staining***

We examined the apical region of left ventricular myocardium and prepared 11 slides stained with H.&E., Masson’s trichrome stains, and immunohistochemistry per section. The monoclonal antibodies against human desmoglein-2, desmocollin-2, plakophilin-2, plakoglobin and desmoplakin which constitute the immunoassay system used in this study were prepared. These monoclonal antibodies belong to the IgG1 subclass. Immunohistochemical reactions using an indirect immunoperoxidase method were obtained as described previously (1). Serial 4-μm-thick sections of paraffin-embedded specimens were dewaxed and immunostained by the streptavidin-biotin method using a DAKO LSAB, according to the manufacturer’s instruction, as reported previously (2). Briefly, the first step involved the inhibition of intrinsic peroxidase activity by the addition of 0.3% hydrogen peroxidase in phosphate-buffered saline. Nonspecific binding was blocked with normal goat serum. As a primary antibody, the monoclonal antibody described above was incubated with the sections for 48 h at 4°C. Diaminobenzidine (Sigma-Aldrich) was used as the chromogen. The presence of immunoreactive desmosome proteins was assessed by light microscopy.

***Amplicon sequence analysis***

The genomic DNA was extracted from peripheral blood of the patient using QIAAmp DNA mini kit (QIAGEN). We prepared genomic DNA library using Ion AmpliSeq Library Kit and Ion Ampliseq Cardiovascular Research Panel (10,430 PCR amplicons covering 404 genes known to harbor mutations affecting cardiovascular function), and sequence run was performed using Ion PGM with 318 Chips. Sequencing data were analyzed using TorrentSuite (version 5.2.2, Life Technologies). Variants with low quality score less than 30 or with low read depth less than 30 were excluded. Among the identified 559 exonic variants, synonymous mutations without amino acid changes were excluded. Variants were classified to be benign when present in 1000 genomes database (3), ESP 6500 database (4, 5), Human Genetic Variation Database (HGVD) (6, 7), or in ExAC database (8), with an allele frequency more than 0.01. After filtering, 13 heterozygous nonsynonymous variants and 1 homozygous stop-gain variant were identified. The stop-gain mutation (c.C355T, p.R119X) in *DSG2* was identified in ExAC database with allele frequency of 0.00002524 but not reported in either 1000 genomes database, ESP 6500, HGVD.

***Chemically defined differentiation of human iPSC-CMs***

The human iPSCs were differentiated into human iPSC-CMs using a chemically defined protocol (9). The culture medium was exchanged for differentiation medium that contained RPMI 1640 medium (ThermoFisher Scientific, USA), recombinant human albumin (Sigma-Aldrich) and L-ascorbic acid 2-phosphate (Sigma-Aldrich). iPSCs were treated with CHIR99021 (LC Laboratories, USA) (day 0 to 2), Wnt-C59 (Selleck Chemicals, USA) and XAV-939 (Cayman, USA) (day 2 to 4). When replating, iPSC-CMs were dissociated with 0.25% Trypsin-EDTA (Gibco, USA) on day 14 after differentiation and reseeded on Gelatin-coated plates.

***Single strand annealing assays***

Targeted genomic sequences of human *DSG2* were cloned into pCAG-EGxxFP vector (10) encoding tandem truncated EGFP gene with overlapped sequence separated by the cloning site. pCAG-EGxxFP vector and pX459 vector (11) encoding SpCas9 and the gRNAs were transfected into HEK293T cells pre-seeded in Greiner CELLSTAR 96-well plate (1 × 10^4^ cells/well). The fluorescent images of EGFP were obtained by high-content image analysis (IN Cell Analyzer 6000; GE) 48 h after transfection, and quantitatively analyzed using IN Cell Developer Toolbox (GE). A total of 36 nonoverlap images (9 images per well) were obtained from each sample in one experiment using a 10×/0.45NA Nikon lens.

***Transfection of genome editing components into human iPSCs.***

Dissociated iPSCs were washed twice with OPTI-MEM (Gibco, USA). After centrifuge at 800 × g for 5 min, iPSCs were suspended with OPTI-MEM at 1 × 10^5^ cells / 100 μl and mixed with 5 μg pX459 vector encoding sgRNA against *DSG2* sequence and 5 μg repair template DNA (pCR blunt II vector). Electroporation was performed using NEPA 21 electroporator (Poring pulse: pulse voltage 125 V, pulse width 5 ms, pulse interval 50 ms, pulse number, 2. Transfer pulse: pulse voltage 20 V, pulse width 20 ms, pulse interval 50 ms, pulse number, 5). Electroporated iPSCs were incubated in 24-well plates for 3 days, then 250 cells were passaged to P3.5 dish. After 7 days, generated colonies were picked up into 96-well plates in duplicate. After immunostaining using anti-desmoglein-2 antibody, selected iPSC clones were further expanded and genotyped.

***Transmission electron microscopy***

iPSC-CMs were observed using a TEM (H-7650; Hitachi Co., Tokyo, Japan). To prepare specimens for TEM, cells were fixed with 2.5% glutaraldehyde for 2 h. After fixation with 1% osmium tetraoxide for 90 min, the cells were dehydrated through a graded series of ethanol (50–100%) and propylene oxide and embedded in epoxy resin. Ultrathin sections were cut using an ultramicrotome (Ultracut E; Reichert-Jung, Vienna, Austria) and stained with uranyl acetate and lead citrate.

***Western blotting***

The protein concentration of each fractionated lysate was determined when required by BCA Protein Assay Kit (Thermo). Lysate samples were mixed with 4 × Laemmli sample buffer (BioRad) with mercaptoethanol (2.5%). Antibodies were diluted by 3% nonfat milk. After blocking with 3% nonfat milk for 1 h, the transferred membrane was incubated with a primary antibody at 4°C overnight and with secondary antibody at room temperature for 30 min. The membrane signals were detected by chemiluminescence using ECL prime reagent (GE). Western blotting data were obtained using ImageQuant LAS 4000 (GE) system.

***Immunofluorescence***

Cardiomyocytes were fixed with 4% PFA for 15 min at room temperature. After fixation, cells were permeabilized with 0.5% Triton-X 100 for 20 min, then incubated for 1 h with 1% BSA in PBS at room temperature. After blocking, cells were incubated with primary antibodies diluted with 1% BSA in PBS for overnight at 4°C. For secondary reaction, an Alexa 488-, 568- or 647- labeled secondary antibody (Invitrogen) was used.

***Measurement of the electro activity of the iPSC-CMs using MEA***

The multielectrode array (MEA) data acquisition system (USB-ME64-System; Multi Channel Systems, Germany) was used for recording field potentials of CMs. After dissociation, the cells were re-suspended in serum-supplemented culture medium containing 40% high glucose DMEM (Sigma-Aldrich), 40% IMDM (Sigma-Aldrich), 20% fetal bovine serum (FBS; Gibco, USA), 1% minimum essential medium non-essential amino acid solution (Sigma-Aldrich), and 0.1% penicillin-streptomycin (Gibco, USA), 0.5% L-glutamine (Sigma-Aldrich), and plated on the MEA chips at a density of 1 × 10^6^ cells cm^-2^. Electrical signals were recorded at day14 after replating of iPSC-CMs. The data were collected and processed using MC_Rack (Multi Channel Systems) (12, 13). Corrected QT interval was calculated by normalization to the beating rate using Fridericia correction formula as previously described (12). Conduction velocity obtained from the MEA data were processed according to the previously reported method (14). The minimum of first derivative plot of the raw data was calculated to obtain the local activation time (LAT) for each single electrodes. Conduction velocity is calculated by using the LAT and the electrode position, and the isochronal map was generated by using the Matlab plotting function (Matlab, USA).

***Generation and purification of AAV***

To generate AAV2, HEK293T cells were transfected with pAAV vector encoding C-terminally HA-tagged DSG2, pHelper vector and pRC2-mi342 vector (AAVpro Helper Free System; TaKaRa, Japan) using calcium phosphate transfection (CalPhos Mammalian Transfection Kit; TaKaRa). Seventy-two h after transfection, HEK293T cells were detached by addition of 1/80 volume of 0.5M EDTA (pH 8.0), then pelleted via low-speed centrifugation (2000 × g for 10 min). Cell pellet was lysed with AAV Extraction Solution A and centrifuged (9000 × g for 10 min). AAV Extraction Solution B was added to the collected to supernatant and stored at -80°C. Collected AAV generated from HEK293T cells was purified using AAVpro Purification Kit (TaKaRa), and viral titer was calculated using AAV Titration Kit (TaKaRa). Transduction efficiency of AAV2-mediated gene delivery in the iPSC-CMs was quantitatively evaluated by high-content image analysis (IN Cell Analyzer 6000; GE) using IN Cell Developer Toolbox (GE)(15).

**Reference**

1 Hasegawa, K., Fujiwara, H., Doyama, K., Mukoyama, M., Nakao, K., Fujiwara, T., Imura, H. and Kawai, C. (1993) Ventricular expression of atrial and brain natriuretic peptides in dilated cardiomyopathy. An immunohistocytochemical study of the endomyocardial biopsy specimens using specific monoclonal antibodies. *Am J Pathol*, **142**, 107-116.

2 Hatanaka, K., Li, X.A., Masuda, K., Yutani, C. and Yamamoto, A. (1995) Immunohistochemical localization of C-reactive protein-binding sites in human atherosclerotic aortic lesions by a modified streptavidin-biotin-staining method. *Pathol Int*, **45**, 635-641.

3 Genomes Project, C., Auton, A., Brooks, L.D., Durbin, R.M., Garrison, E.P., Kang, H.M., Korbel, J.O., Marchini, J.L., McCarthy, S., McVean, G.A. *et al.* (2015) A global reference for human genetic variation. *Nature*, **526**, 68-74.

4 Fu, W., O'Connor, T.D., Jun, G., Kang, H.M., Abecasis, G., Leal, S.M., Gabriel, S., Rieder, M.J., Altshuler, D., Shendure, J. *et al.* (2013) Analysis of 6,515 exomes reveals the recent origin of most human protein-coding variants. *Nature*, **493**, 216-220.

5 Tennessen, J.A., Bigham, A.W., O'Connor, T.D., Fu, W., Kenny, E.E., Gravel, S., McGee, S., Do, R., Liu, X., Jun, G. *et al.* (2012) Evolution and functional impact of rare coding variation from deep sequencing of human exomes. *Science*, **337**, 64-69.

6 Narahara, M., Higasa, K., Nakamura, S., Tabara, Y., Kawaguchi, T., Ishii, M., Matsubara, K., Matsuda, F. and Yamada, R. (2014) Large-scale East-Asian eQTL mapping reveals novel candidate genes for LD mapping and the genomic landscape of transcriptional effects of sequence variants. *PLoS One*, **9**, e100924.

7 Higasa, K., Miyake, N., Yoshimura, J., Okamura, K., Niihori, T., Saitsu, H., Doi, K., Shimizu, M., Nakabayashi, K., Aoki, Y. *et al.* (2016) Human genetic variation database, a reference database of genetic variations in the Japanese population. *J Hum Genet*, **61**, 547-553.

8 Lek, M., Karczewski, K.J., Minikel, E.V., Samocha, K.E., Banks, E., Fennell, T., O'Donnell-Luria, A.H., Ware, J.S., Hill, A.J., Cummings, B.B. *et al.* (2016) Analysis of protein-coding genetic variation in 60,706 humans. *Nature*, **536**, 285-291.

9 Burridge, P.W., Matsa, E., Shukla, P., Lin, Z.C., Churko, J.M., Ebert, A.D., Lan, F., Diecke, S., Huber, B., Mordwinkin, N.M. *et al.* (2014) Chemically defined generation of human cardiomyocytes. *Nat Methods*, **11**, 855-860.

10 Mashiko, D., Fujihara, Y., Satouh, Y., Miyata, H., Isotani, A. and Ikawa, M. (2013) Generation of mutant mice by pronuclear injection of circular plasmid expressing Cas9 and single guided RNA. *Sci Rep*, **3**, 3355.

11 Ran, F.A., Hsu, P.D., Wright, J., Agarwala, V., Scott, D.A. and Zhang, F. (2013) Genome engineering using the CRISPR-Cas9 system. *Nat Protoc*, **8**, 2281-2308.

12 Li, J., Minami, I., Shiozaki, M., Yu, L., Yajima, S., Miyagawa, S., Shiba, Y., Morone, N., Fukushima, S., Yoshioka, M. *et al.* (2017) Human Pluripotent Stem Cell-Derived Cardiac Tissue-like Constructs for Repairing the Infarcted Myocardium. *Stem Cell Reports*, **9**, 1546-1559.

13 Li, J., Zhang, L., Yu, L., Minami, I., Miyagawa, S., Horning, M., Dong, J., Qiao, J., Qu, X., Hua, Y. *et al.* (2020) Circulating re-entrant waves promote maturation of hiPSC-derived cardiomyocytes in self-organized tissue ring. *Commun Biol*, **3**, 122.

14 Meiry, G., Reisner, Y., Feld, Y., Goldberg, S., Rosen, M., Ziv, N. and Binah, O. (2001) Evolution of action potential propagation and repolarization in cultured neonatal rat ventricular myocytes. *J Cardiovasc Electrophysiol*, **12**, 1269-1277.

15 Kohama, Y., Higo, S., Masumura, Y., Shiba, M., Kondo, T., Ishizu, T., Higo, T., Nakamura, S., Kameda, S., Tabata, T. *et al.* (2020) Adeno-associated virus-mediated gene delivery promotes S-phase entry-independent precise targeted integration in cardiomyocytes. *Sci Rep*, **10**, 15348.

16 Kolegraff, K., Nava, P., Laur, O., Parkos, C.A. and Nusrat, A. (2014) Characterization of full-length and proteolytic cleavage fragments of desmoglein-2 in native human colon and colonic epithelial cell lines. *Cell Adhesion & Migration*, **5**, 306-314.

**Supplementary Figure Legends**

Supplementary Figure 1

1. Premature ventricular contraction and a wide QRS beat were observed in the electrocardiogram of the patient (II-1, 21 y.o.). Electrocardiogram findings of the parents (I-1, 61 y.o. and I-2, 55 y.o.) were within normal range.
2. Result of echocardiogram parameter of Fig 1E. LVDd, left ventricular end-diastolic diameter; LVDs, left ventricular end-systolic diameter; IVSd, intraventricular septal end-diastolic diameter; PWd, posterior wall end-diastolic diameter; LVEF, left ventricular ejection fraction.
3. Masson trichrome-stained LV myocardium obtained from the patient (II-1). Myocyte loss with replacement and interstitial fibrosis are diffusely observed in the LV. Scale bar = 200 μm.

Supplementary Figure 2

1. Pt-, HDR-, and R119X-iPSCs were fixed and immunostained with the indicated pluripotent markers. Nuclei were detected by Hoechst staining. Scale bar: 100 µm.
2. The results of karyotype analysis using Pt-, HDR- and R119X-iPSCs.
3. Cleaving activities of each gRNA were evaluated by single-strand annealing assay (**p* < 0.0001 vs control sample (HEK293T cells transfected with Cas9 without gRNA), n = 4). Data are reported as the mean ± SD.
4. R119X-iPSCs were transfected with pX459 encoding gRNA #2. After puromycin selection, genomic DNA was extracted from the iPSC clones. The targeted sequence in *DSG2* was amplified by PCR, cloned into a plasmid vector, and sequenced. Representative data from the five clones with NHEJ are shown. The PAM sequence targeted by gRNA #2 is highlighted in red. The inserted base pair is highlighted in blue.
5. Genomic DNA and amino-acid sequence of WT and C355T alleles are shown. In HDR-iPSCs, a synonymous sequence was introduced into the HDR allele to prevent Cas9-mediated re-cleavage. The insertion mutation 297dupT was introduced via NHEJ in the allele opposite to that encoding the frameshift mutation and protein termination at amino-acid residue 105. The C355T mutation is highlighted in red. The substituted residues in the synonymous sequence and repaired C residue in HDR-iPSCs are highlighted in blue.
6. Whole-cell lysates were extracted from R119X-, HDR- and Ctrl-iPSC-CMs on day 14 after differentiation and analyzed by western blotting using the indicated antibodies. Monoclonal anti-desmoglein-2 antibody (AH12.2) that recognizes the first extracellular domain of desmoglein-2 (16) was used. The truncated protein band caused by R119X mutation was not detected in R119X-iPSC-CMs.

Supplementary Figure 3

1. Relative expression in R119X- and HDR-iPSC-CMs normalized by α-Actinin (Fig. 5B) (***p* < 0.001 vs control sample (HDR-iPSC-CMs), desmoglein-2; n = 7, desmocollin-2; n = 6, plakophilin-2; n = 6, plakoglobin; n = 6, desmoplakin; n = 4). Data are reported as the mean ± SD.
2. Whole-cell lysates were extracted from R119X- and HDR-iPSC-CMs on day 14 and analyzed by western blotting using the indicated antibodies (upper). Relative expression in R119X- and HDR-iPSC-CMs were normalized by α-Actinin (N-cadherin; n = 4, connexin 43; n = 4). Data are reported as the mean ± SD (lower).
3. Fourteen days after differentiation, both R119X- and HDR-iPSC-CMs were replated into 24-well plates containing pillars precoated with laminin for continuous observation of the 2-dimensional structures. Bright fields of the replated iPSC-CMs were sequentially observed on days 20, 24, 27, 32, and 38 (days 6, 10, 13, 18, and 24 after replating). Dotted lines shown in the bright field images in R119X-iPSC-CMs on days 32 and 38 indicate the periphery of the cardiomyocytes. Scale bar: 100 μm.

Supplementary Figure 4

1. iPSC-CMs transduced with AAV2-EGFP (1.04 × 10^4^ or 2.60 × 10^4^ vg/cell) were fixed and immunostained with anti-troponin T antibody 5 days after transduction. Nuclei were detected by Hoechst staining. Scale bar: 100 µm. NTD indicates the non-transduction control. The proportion of GFP-positive cardiomyocytes was quantitatively analyzed using high-content imaging. Transduction of AAV2 at 1.04 – 2.60 x 10^4^ vg/cell resulted in approximately 70 – 90% GFP-positive iPSC-CMs.
2. Genomic DNA and amino-acid sequence in Pat-, Pat-NHEJ-, Cm- and Cm-NHEJ-iPSCs are shown. Homozygous frameshift mutations (298delG, G100fsX111 and 298dupG, G100fsX105) were introduced into Pat-NHEJ-iPSCs via NHEJ (upper). Homozygous frameshift mutations (298dupG, G100fsX105) were introduced into Cm-NHEJ-iPSCs via NHEJ (lower). The C355T mutation, the introduced 298delG and 298dupG mutations are highlighted in red.
3. Scheme of genomic sequence of *DSG2* (upper) and the translated amino-acid sequence of desmoglein-2 (lower) in Pat-, Pat-NHEJ-, Cm- and Cm-NHEJ-iPSCs.

Supplementary Figure 5

1. Pat-, Pat-NHEJ-, Cm- and Cm-NHEJ-iPSC-CMs replated in 96-well plates on day 14 were fixed and immunostained on day 20 with the indicated antibodies. Scale bar: 50 μm.
2. Whole-cell lysates were extracted from Pat-, Pat-NHEJ-, Cm- and Cm-NHEJ-iPSC-CMs and analyzed by western blotting using the indicated antibodies.

Supplementary Table 1

PCR primers and DNA oligonucleotides used in this study.

Supplementary Table 2

Antibodies and reagents used in this study.

Supplementary Video 1

Micro motion of the SOTR generated from R119X-iPSC-CMs compressed by cantilever beam. Under increasing compression force, the SOTR suddenly broke.

Supplementary Video 2

Micro motion of the SOTR generated from R119X-iPSC-CMs compressed by cantilever beam.

Supplementary Video 3

Micro motion of the SOTR generated from HDR-iPSC-CMs compressed by cantilever beam.
